# Diagnostic challenges of giant cervical pilomatrixoma mimicking malignancy: case report and focused literature review

**DOI:** 10.3389/fmed.2026.1758413

**Published:** 2026-02-03

**Authors:** Horatiu Urechescu, Marius Pricop, Flavia Zara, Raluca Maria Closca, Felicia Streian

**Affiliations:** 1 Department of Oral and Maxillo-Facial Surgery, Victor Babes University of Medicine and Pharmacy, Timisoara, Romania; 2 Department of Microscopic Morphology, Victor Babes University of Medicine and Pharmacy, Timisoara, Romania; 3 Angiogenesis Research Center, Victor Babes University of Medicine and Pharmacy, Timisoara, Romania; 4 Service of Pathology, Emergency City Hospital, Timisoara, Romania

**Keywords:** benign skin adnexal tumor, case report, cervical mass, core needle biopsy, giant pilomatrixoma, surgical excision

## Abstract

Pilomatrixoma is a benign skin adnexal tumor arising from the hair-follicle matrix. While typically small and slowly growing, rare “giant” variants (>5 cm)—especially in the cervical region—may clinically and radiologically mimic malignant masses, posing a significant diagnostic challenge. We report a case of a 29-year-old male presenting with a solitary, firm, subcutaneous mass (~6 cm) in the left anterior cervical region, which enlarged rapidly over 6 months without pain or systemic symptoms. Contrast-enhanced CT revealed a well-defined, heterogeneous subcutaneous lesion without muscular or vascular invasion. Core needle biopsy (CNB) supported a diagnosis of pilomatrixoma. The mass was completely excised under IV sedation and local anesthesia. Histopathology confirmed classic pilomatrixoma features (basaloid cells, shadow cells, foreign-body giant cell reaction). At one-year follow-up the patient was disease-free, and scar outcome was excellent. This case underscores the importance of including giant pilomatrixoma in the differential diagnosis of large cervical soft-tissue masses. Histopathological examination remains essential for definitive diagnosis, while CNB can guide preoperative planning. Complete surgical excision yields excellent outcomes with minimal morbidity, avoiding overtreatment for suspected malignancy.

## 1 Introduction

Pilomatrixoma (pilomatricoma) is a benign adnexal neoplasm arising from the hair-follicle matrix and represents approximately 0.1–0.12% of all skin tumors, with a slight female predominance and a predilection for the head and facial region (1–3). Clinically, it presents as a firm, slow-growing, subcutaneous nodule and typically measures less than 3 cm. Although most cases follow a characteristic benign course, some presentations may deviate from the classic pattern.

“Giant” pilomatrixomas, defined as lesions exceeding 5 cm in diameter, are uncommon and demonstrate distinct clinical behavior compared with classic forms. Reported lesions have reached sizes up to 14–15 cm, and recent literature highlights their tendency to occur more frequently in adults and within the head and neck region (4–7). Their atypical size, rapid growth, and occasional ulceration frequently raise suspicion for malignant disease. In the cervical area in particular, the differential diagnosis is broad and includes lymphadenopathy, sarcomas, cystic lesions, adnexal tumors, and pilomatrix carcinoma (7–9).

These atypical features can create significant diagnostic uncertainty. Imaging may not reliably distinguish giant pilomatrixoma from more aggressive pathology, and while fine-needle aspiration cytology can be suggestive when basaloid cells and shadow cells are identified, sampling limitations may complicate interpretation. Core needle biopsy (CNB), by providing a more representative specimen, may improve diagnostic confidence, although definitive diagnosis still relies on histopathological examination.

Recognizing this unusual variant is important to avoid unnecessary patient anxiety and overtreatment. In this report, we describe a rare case of a giant pilomatrixoma of the neck, emphasizing the diagnostic work-up, the contribution of CNB, and key considerations in distinguishing this entity from malignant disease.

## 2 Case description

### 2.1 Clinical findings

A 29-year-old male patient presented with a solitary mass in the left anterior cervical region. He could not recall the exact time of onset, noting that the lesion had been present for an extended period but had undergone a noticeable and rapid increase in size over the preceding 6 months. Although the enlargement was not associated with pain, discharge, or systemic symptoms, the patient reported significant concern regarding the recent rapid growth.

**Figure 1:**
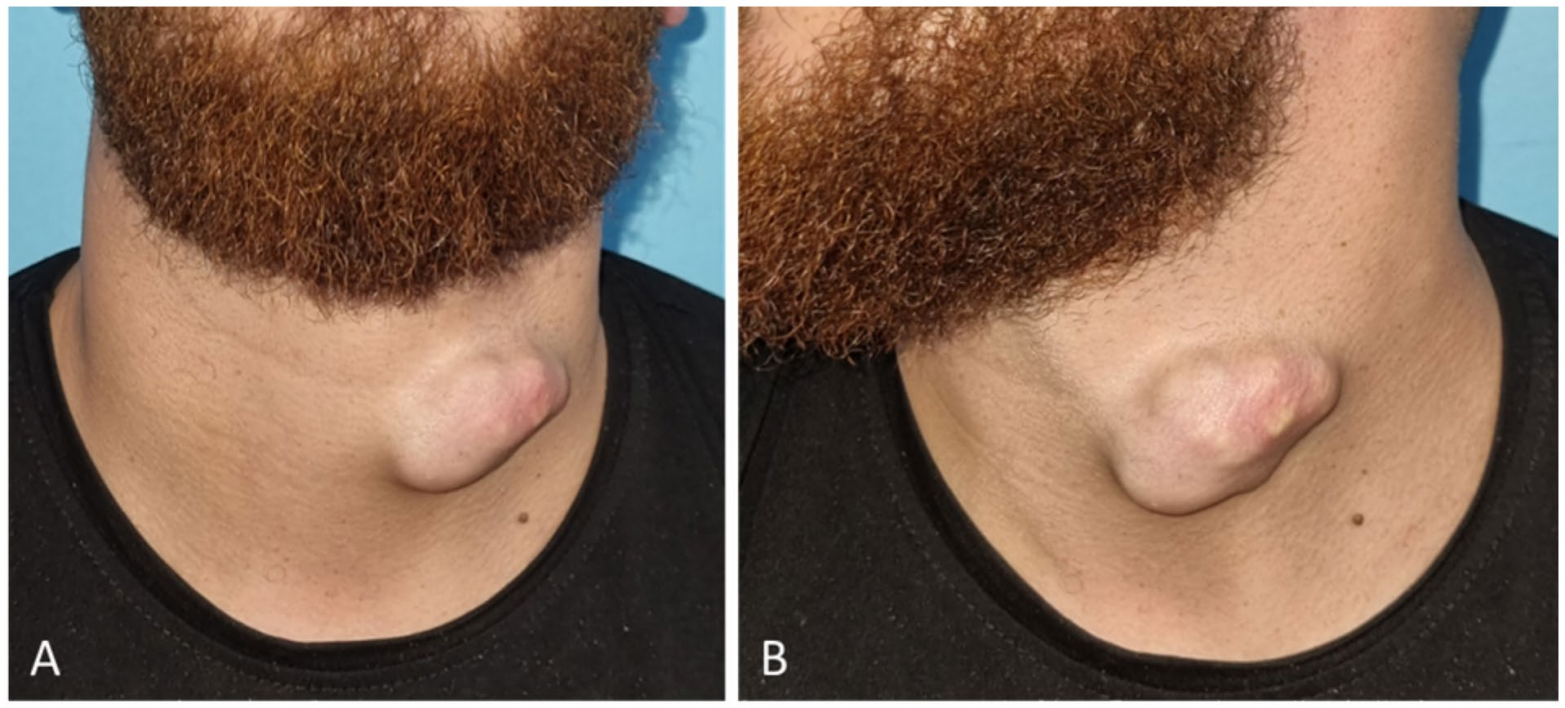
Preoperative clinical appearance of the cervical mass. **(A)** Frontal view showing the left anterior cervical subcutaneous mass. **(B)** Lateral view demonstrating its contour and subcutaneous location.

On examination, a well-circumscribed, dome-shaped subcutaneous mass measuring approximately 6 cm in greatest dimension was identified (Figures 1A,B). The overlying skin displayed a smooth surface with mild discoloration, without erythema, ulceration, or inflammatory changes. The lesion was firm and non-tender on palpation and demonstrated partial mobility over deeper structures, with slight adherence to the overlying skin. No cervical lymphadenopathy was detected, and the remainder of the head and cervical examination was unremarkable. The patient had no significant past medical or surgical history, took no regular medications, and reported no relevant family or genetic conditions. Social and occupational history was non-contributory (Table 1).

**Table 1:** Clinical timeline of the case.

Time point	Event
Unknown onset	Patient first notices a small, painless subcutaneous mass in the left anterior cervical region (exact timing not recalled).
≈ 6 months before presentation	Rapid enlargement of the mass begins; patient becomes concerned due to sudden growth.
Initial clinical evaluation	Physical examination reveals a firm, ~6 cm subcutaneous cervical mass with mild skin discoloration; no pain, ulceration, or lymphadenopathy.
Diagnostic imaging (CT)	CT demonstrates a well-defined, heterogeneous subcutaneous lesion adjacent to the sternocleidomastoid muscle without fascial or vascular invasion.
Tissue diagnosis (core needle biopsy)	CNB performed under local anesthesia; samples obtained without complications.
Histopathological analysis of CNB	Findings support pilomatrixoma.
Surgical intervention	En bloc excision of the mass performed under IV sedation and local anesthesia; lesion removed intact; no intraoperative complications.
Immediate postoperative period	Wound closure achieved with good alignment; uncomplicated recovery.
Final histopathology	Confirms pilomatrixoma with classic features; no evidence of malignancy.
1-year follow-up	Excellent cosmetic outcome; well-healed linear cervical scar; no clinical evidence of recurrence.

### 2.2 Diagnostic assessment

#### 2.2.1 Imaging

**Figure 2:**
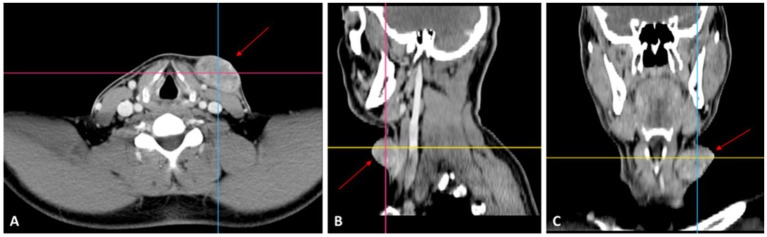
Contrast-enhanced CT evaluation of the lesion. **(A)** Axial image showing a well-defined subcutaneous mass (red arrow). **(B)** Sagittal image demonstrating confinement to the subcutaneous plane (red arrow). **(C)** Coronal image showing displacement without invasion of adjacent structures (red arrow).

Contrast-enhanced computed tomography (CT) of the cervical region revealed a well-defined, macronodular soft-tissue mass located in the left anterior subcutaneous compartment (Figures 2A–C). The lesion measured approximately 5.5 × 4.0 × 3.0 cm and demonstrated a heterogeneous internal structure following contrast administration, without areas of central necrosis. It was tangent to the anterior border of the left sternocleidomastoid muscle but showed no evidence of infiltration into the underlying musculature or deeper fascial planes. The adjacent vascular structures were preserved, without signs of encasement or compression, and no cervical lymphadenopathy was observed.

The mass exhibited smooth and well-demarcated margins, consistent with a subcutaneous origin. Although mild internal density variations were present, no overt intralesional calcifications were identified on CT. The lesion’s size, subcutaneous location, and heterogeneous enhancement were suggestive of a benign skin adnexal tumor with atypical features; however, the absence of deep invasion or nodal involvement argued against an aggressive malignant process.

Although ultrasound represents a valuable, non-invasive first-line modality for the evaluation of superficial soft-tissue masses, it was not performed in this case due to technical limitations at the time of initial assessment; therefore, contrast-enhanced CT was selected to accurately assess lesion extent and its relationship with adjacent cervical structures.

Based on the CT appearance, the differential diagnosis included pilomatrixoma, epidermoid or dermoid cyst with secondary changes, benign adnexal tumors, and, less likely, soft-tissue sarcomas or metastatic lymphadenopathy, particularly given the reported recent rapid growth.

#### 2.2.2 Core needle biopsy

Given the lesion’s recent rapid enlargement and the nonspecific appearance, a core needle biopsy (CNB) was performed to obtain tissue for histopathological evaluation. CNB was chosen as it provides a larger and more structurally representative specimen than fine-needle aspiration, improving diagnostic reliability for subcutaneous soft-tissue and adnexal lesions.

The biopsy was carried out under sterile conditions and local anesthesia using a spring-loaded automated core biopsy device, targeting the most representative area of the mass by direct palpation. Several tissue cores were obtained without complication, fixed in 10% (v/w) neutral buffered formalin and sent to the Department of Pathology for a histopathological examination.

**Figure 3:**
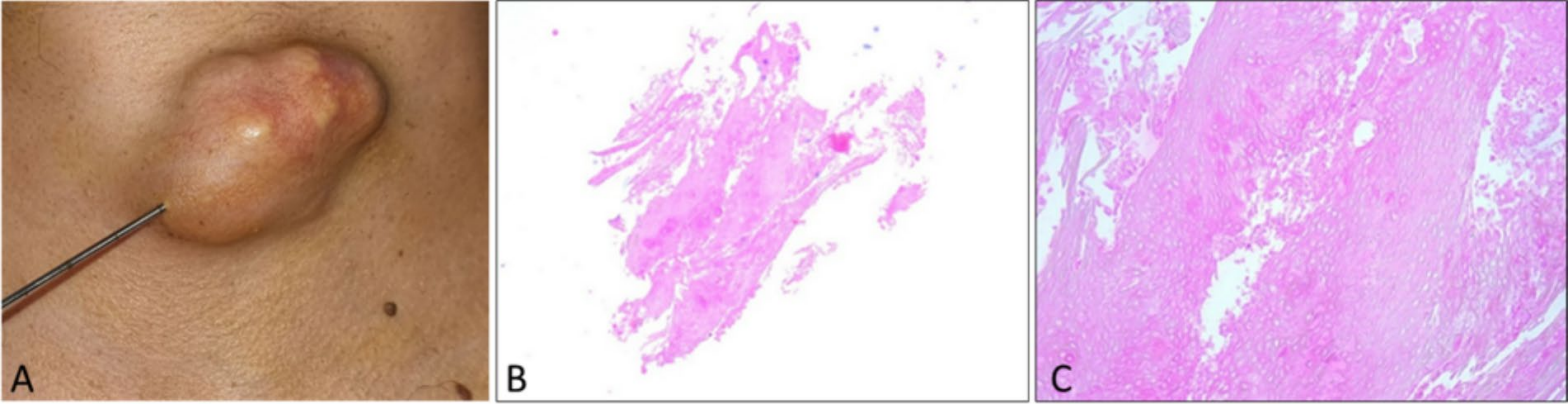
Core needle biopsy: **(A)** Clinical view during core needle biopsy. **(B)** Low-power H&E section (5×). **(C)** Higher-power H&E section (20×).

Four-micrometer-thick serial sections were prepared for the diagnosis from paraffin blocks, using morphological Hematoxylin–Eosin staining. The microscopic examination revealed an eosinophilic structured matrix, with a few large polygonal cell silhouettes, without obvious nuclei (Figure 3). A presumptive diagnosis of benign skin adnexal tumor originating from hair matrix and cortex was made.

### 2.3 Therapeutic intervention

**Figure 4:**
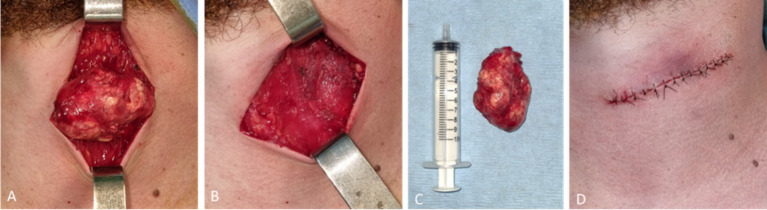
Intraoperative findings and excised specimen. **(A)** Exposure of the well-circumscribed subcutaneous mass. **(B)** Surgical bed after complete excision. **(C)** Gross specimen demonstrating lobulated morphology. **(D)** Immediate postoperative skin closure.

The patient underwent surgical excision of the cervical mass under intravenous sedation complemented with local anesthesia. A transverse cervical incision was made over the most prominent aspect of the lesion, followed by subplatysmal dissection to expose the mass. Intraoperatively, a well-circumscribed, lobulated tumor was visualized within the subcutaneous plane of the left anterior cervical region (Figure 4A). The lesion was firm, encapsulated, and clearly demarcated from the surrounding tissues, with no evidence of infiltration into the underlying musculature or adjacent neurovascular structures.

Sharp and blunt dissection allowed for complete mobilization of the mass, which was excised en bloc without rupture (Figure 4B). The operative field revealed intact deeper fascial and muscular layers. Grossly, the specimen was solid, lobulated, and heterogeneous (Figure 4C). After achieving meticulous hemostasis, layered closure was performed, resulting in an aligned and cosmetically acceptable incision line (Figure 4D).

The procedure was completed without intraoperative complications.

The harvested tissue, fixed in 10% (v/w) neutral buffered formalin, was sent to the Department of Pathology for a histopathological examination. The gross examination of the harvested specimen revealed an oval piece of 5.5 × 3.5 × 3 cm, with thick brown capsule, and smooth surface. In section, an inhomogeneous appearance was identified, with whitish and gray areas, some of increased consistency. A complete section was performed on the long axis which was processed in paraffin in two complementary fragments.

**Figure 5:**
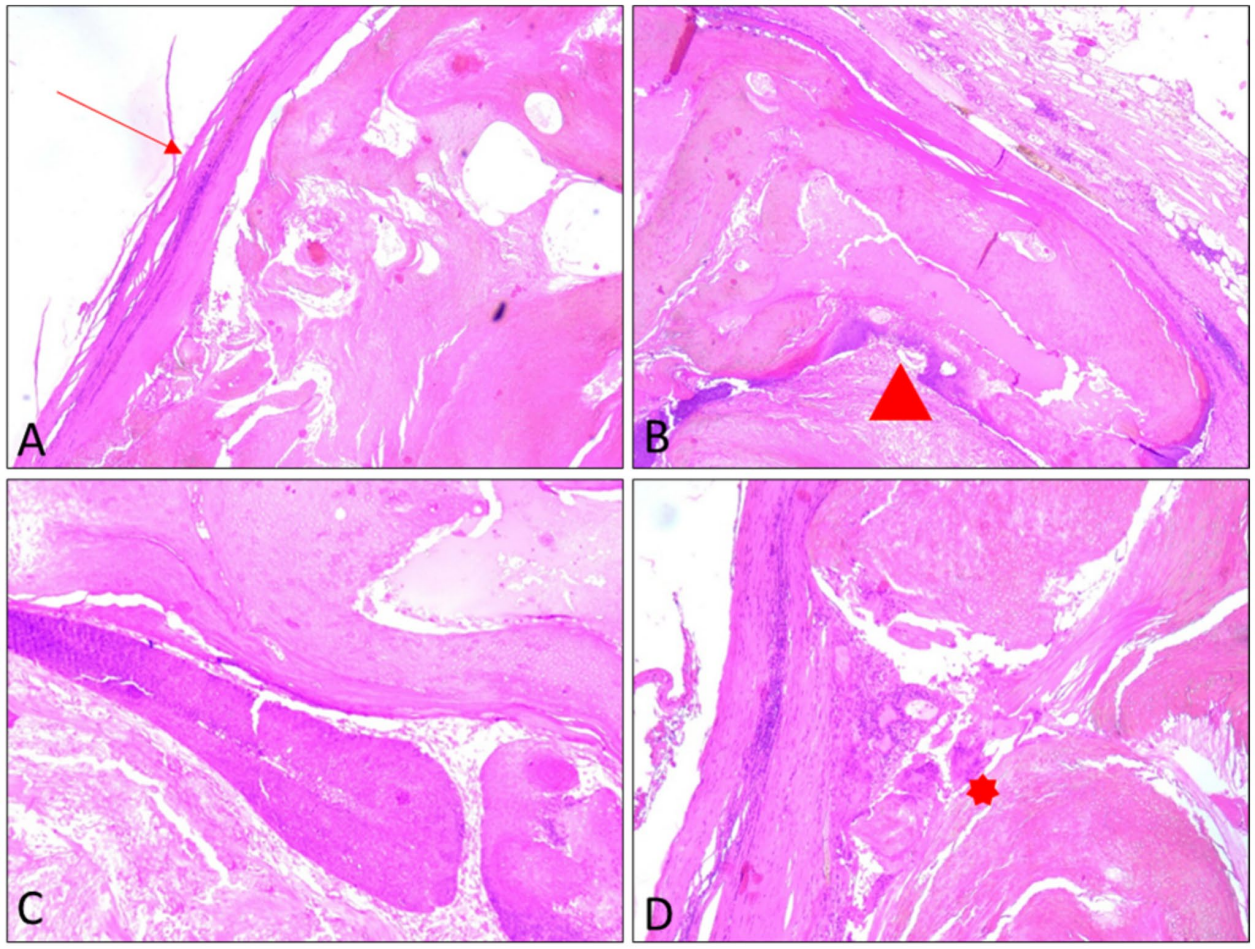
Histopathological features of the excised lesion: **(A)** Low-power view (5×) showing a fibrous capsule (arrow). **(B)** Low-power view (5×) illustrating shadow (ghost) cells (arrowhead). **(C)** Higher-power view (20×) showing basaloid cell areas. **(D)** Higher-power view (20×) demonstrating granulomatous reaction with multinucleated giant cells (asterisk).

Four-micrometer-thick serial sections were prepared for the diagnosis from paraffin blocks, using morphological Hematoxylin–Eosin staining. The microscopic examination revealed a benign proliferation circumscribed by a fibrous capsule. The tumor presented a lobulated appearance and islands of basaloid cells with abrupt keratinization, without an intermediate granular layer, as well as numerous ghost cells. The basaloid cells showed mitotic activity with typical mitoses, and the intermediate cells had intensely eosinophilic cytoplasm and pyknotic nuclei. The tumor presented an inflammatory response with multiple foreign body giant cells, most likely to keratin filaments (Figure 5). Based on these morphological aspects, the diagnosis of pilomatrixoma was established.

### 2.4 Follow-up and outcomes

**Figure 6:**
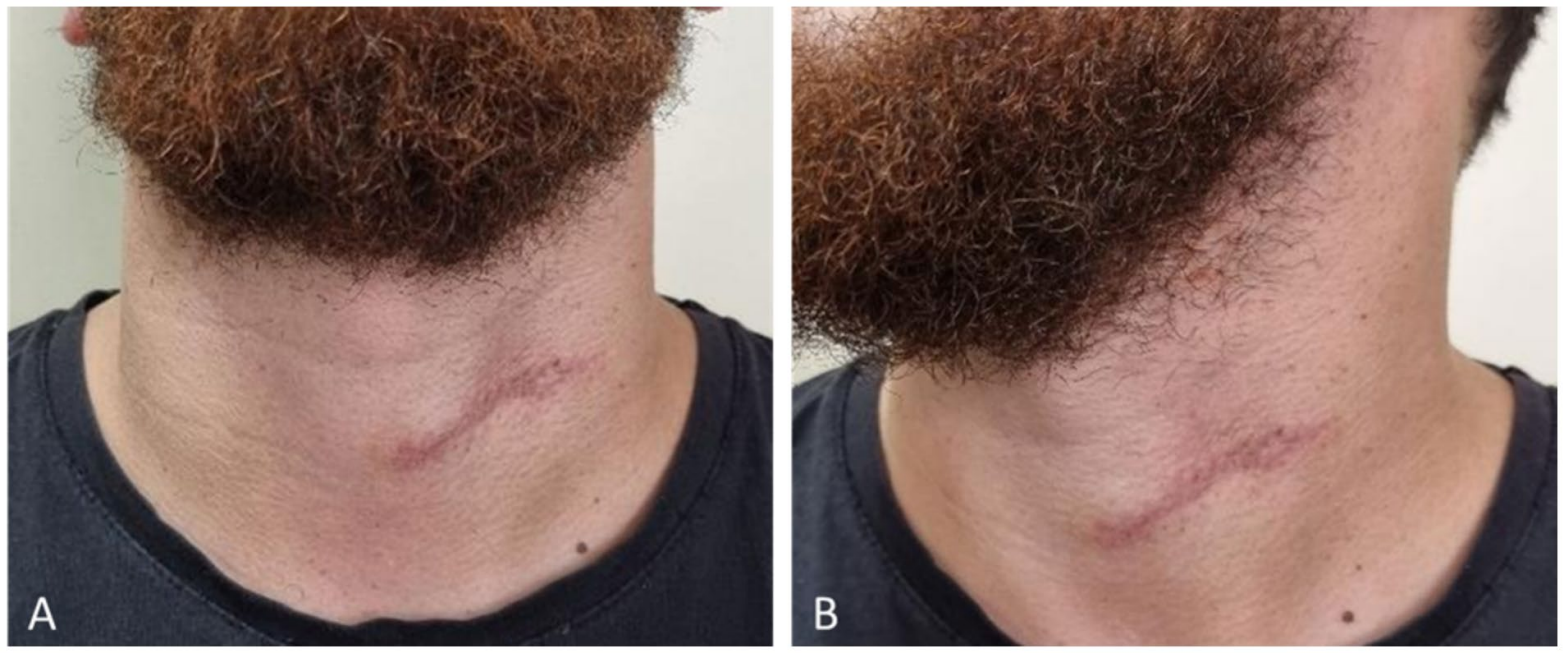
One-year postoperative appearance. **(A)** Frontal view showing a well-healed linear scar. **(B)** Lateral view demonstrating a mature, flat postoperative scar.

At the one-year postoperative evaluation, the patient demonstrated an excellent clinical outcome with no evidence of recurrence. The surgical site had healed well, displaying a fine, linear cervical scar consistent with normal postoperative maturation (Figures 6A,B). The scar was flat, soft, and asymptomatic, without hypertrophy, contracture, or pigmentary alteration. The patient reported full satisfaction with the esthetic result and experienced no functional limitations or local discomfort.

No palpable masses, induration, or regional lymphadenopathy were detected on examination. Overall, the postoperative course was uneventful, and no evidence of disease recurrence was observed at the latest follow-up.

## 3 Discussion

Giant pilomatrixoma is an uncommon clinical variant of an otherwise small benign adnexal tumor and can present a considerable diagnostic challenge when arising in the cervical region. Their rapid enlargement, firm consistency, and heterogeneous appearance frequently lead clinicians to suspect malignant or metastatic disease. Although pilomatrixoma has been widely reported in the literature, most publications describe small, typical lesions. Clinically comparable giant pilomatrixomas involving the cervical and parotid–upper cervical regions remain exceedingly rare.

Only a small number of giant pilomatrixomas involving the cervical or parotid–upper cervical region have been reported in the literature (Table 2). In nearly all cases, the initial clinical impression favored malignancy—commonly lymphoma, metastatic lymphadenopathy, or parotid cancer—and surgical excision ultimately revealed benign pilomatrixoma (10–16). To better contextualize the present case, we performed a focused narrative review of previously reported giant pilomatrixomas involving the cervical and parotid–upper cervical regions, based exclusively on cases with clearly reported clinical data. The present case mirrors this pattern, as the patient’s rapidly enlarging cervical mass raised substantial concern for an aggressive process.

**Table 2:** Published cases of giant pilomatrixoma in the cervical and parotid–upper cervical region.

Author (year)	Patient age/sex	Cervical/parotid location	Size (cm)	Preoperative diagnosis/mimic	Treatment & outcome
Jha et al. (2022) (10)	35/M	Posterior neck	10 × 10	Suspected lymphoma / malignancy	Complete excision; benign histology; uneventful recovery
Świątek et al. (2025) (11)	57/M	Midline neck	11 × 6 × 4	Suspicion of salivary tumor	Excision; synchronous with Warthin tumor; no recurrence
Mundinger et al. (2011) (12)	53/M	Parotid/post-auricular cervical region	>5	Malignant parotid neoplasm	Complete excision; benign; satisfactory outcome
Cozzi et al. (2011) (13)	Pediatric	Parotid/upper cervical region	>5 (described as “giant”)	Strong suspicion of malignant parotid mass	Excision; benign; no recurrence
Aydın et al. (2014) (14)	Adult	Parotid/upper cervical region	>5	Parotid mass of unclear nature	Complete excision; benign pilomatrixoma
Koh et al. (2020) (15)	34/F	Right parotid/upper cervical region	6.0 × 5.5 × 1.5	Rapid growth suggesting malignancy	Excision; no recurrence at 6 months
Yuca K. et al., (2004) (16)	65/F	Left preauricular region	6 × 4 cm	Diagnostic difficulty; ulcerated lesion raising concern for malignancy (SCC/BCC)	Complete surgical excision; no recurrence mentioned

Giant pilomatrixoma defined as >5 cm; Parotid-region lesions included due to anatomical continuity with upper cervical soft tissues.

Imaging may assist in lesion characterization but often lacks specificity in giant forms. These tumors may appear as well-defined masses with variable internal density yet may not display the calcifications typically associated with smaller pilomatrixomas (7, 8). Consequently, the radiologic appearance may overlap with branchial cleft cysts, epidermoid or dermoid cysts, adnexal tumors, and soft-tissue sarcomas. Pilomatrix carcinoma, although exceedingly rare, must also be considered when evaluating lesions with rapid growth or atypical features (8, 9).

Tissue sampling is therefore a critical component of evaluation. Fine-needle aspiration (FNAC), while potentially informative, has recognized limitations in pilomatrixoma and may yield nondiagnostic or misleading results (8). Within this limited body of literature, the present case adds value by documenting the role of core needle biopsy in the preoperative assessment of a giant cervical pilomatrixoma mimicking malignancy. In this case, CNB provided sufficient diagnostic information to support pilomatrixoma preoperatively and helped guide appropriate surgical planning.

Complete surgical excision remains the definitive management for both classic and giant pilomatrixoma. Consistent with previously reported cervical cases, the lesion in our patient was well circumscribed and confined to the subcutaneous plane, allowing successful en bloc removal with an excellent functional and cosmetic outcome. Histopathology confirmed classic features of pilomatrixoma, including basaloid cell islands, abrupt keratinization, shadow cells, and foreign-body giant cell reaction, clearly distinguishing it from pilomatrix carcinoma (1, 3, 8).

This case highlights the importance of including giant pilomatrixoma in the differential diagnosis of rapidly enlarging cervical masses. Although rare, its clinical presentation may mimic malignant disease, creating substantial diagnostic uncertainty. Incorporating CNB into the work-up of atypical cervical lesions can facilitate accurate preoperative diagnosis, guide management, and prevent unnecessary aggressive interventions.

While this report adds to the limited number of cervical giant pilomatrixoma cases, its conclusions are inherently constrained by its single-case nature. Larger series would help clarify diagnostic accuracy, imaging characteristics, and optimal management strategies for these uncommon lesions.

## 4 Conclusion

Giant pilomatrixoma is an uncommon clinical entity that should be considered in the differential diagnosis of large, firm, and calcified cervical masses, particularly when rapid growth raises concern for malignancy. Although clinical and radiologic findings may be nonspecific, definitive diagnosis relies on histopathological evaluation demonstrating the characteristic features of pilomatrixoma. Complete surgical excision remains curative, and outcomes are excellent when the lesion is removed with clear margins. Recognition of this rare variant is essential to ensure accurate diagnosis and avoid unnecessary overtreatment.

## 5 Patient perspective

The patient reported significant concern when the neck mass began to grow rapidly, fearing that it could be malignant. The possibility of cancer mentioned during the diagnostic process caused considerable anxiety. After surgery and confirmation of a benign pilomatrixoma, the patient expressed great relief and satisfaction with the outcome. They appreciated the clear communication provided throughout the diagnostic work-up and treatment, and reported full recovery with no functional limitations or cosmetic concerns during follow-up.

## Data Availability

The raw data supporting the conclusions of this article will be made available by the authors, without undue reservation.
